# Repurposing of the Tamoxifen Metabolites to Combat Infections by Multidrug-Resistant Gram-Negative Bacilli

**DOI:** 10.3390/antibiotics10030336

**Published:** 2021-03-22

**Authors:** Andrea Miró-Canturri, Rafael Ayerbe-Algaba, Andrea Vila-Domínguez, Manuel E. Jiménez-Mejías, Jerónimo Pachón, Younes Smani

**Affiliations:** 1 Clinical Unit of Infectious Diseases, Microbiology and Preventive Medicine, University Hospital Virgen del Rocío, 41013 Seville, Spain; amirocan93@gmail.com (A.M.-C.); ayerberafael@gmail.com (R.A.-A.); avila-ibis@us.es (A.V.-D.); mej-mejias@telefonica.net (M.E.J.-M.); 2 Institute of Biomedicine of Seville (IBiS), University Hospital Virgen del Rocío, CSIC, University of Seville, 41013 Seville, Spain; pachon@us.es; 3 Department of Medicine, University of Seville, 41009 Seville, Spain

**Keywords:** tamoxifen, tamoxifen metabolite, bacteria, infection, treatment

## Abstract

The development of new strategic antimicrobial therapeutic approaches, such as drug repurposing, has become an urgent need. Previously, we reported that tamoxifen presents therapeutic efficacy against multidrug-resistant (MDR) *Acinetobacter baumannii*, *Pseudomonas aeruginosa*, and *Escherichia coli* in experimental infection models by modulating innate immune system cell traffic. The main objective of this study was to analyze the activity of N-desmethyltamoxifen, 4-hydroxytamoxifen, and endoxifen, three major metabolites of tamoxifen, against these pathogens. We showed that immunosuppressed mice infected with *A. baumannii*, *P. aeruginosa*, or *E. coli* in peritoneal sepsis models and treated with tamoxifen at 80 mg/kg/d for three days still reduced the bacterial load in tissues and blood. Moreover, it increased mice survival to 66.7% (for *A. baumannii* and *E. coli*) and 16.7% (for *P. aeruginosa*) when compared with immunocompetent mice. Further, susceptibility and time-kill assays showed that N-desmethyltamoxifen, 4-hydroxytamoxifen, and endoxifen exhibited minimum inhibitory concentration of the 90% of the isolates (MIC_90_) values of 16 mg/L, and were bactericidal against clinical isolates of *A. baumannii* and *E. coli*. This antimicrobial activity of tamoxifen metabolites paralleled an increased membrane permeability of *A. baumannii* and *E. coli* without affecting their outer membrane proteins profiles. Together, these data showed that tamoxifen metabolites presented antibacterial activity against MDR *A. baumannii* and *E. coli*, and may be a potential alternative for the treatment of infections caused by these two pathogens.

## 1. Introduction

Anticancer drugs developed to combat breast cancer, such as selective estrogen receptor modulators (SERMs), have been reported to present activity against Gram-positive bacteria [1]. Clomiphene has demonstrated efficacy against *Enterococcus faecium* and *Staphylococcus aureus* through inhibiting undecaprenyl diphosphate synthase (UPPS), an enzyme involved in the synthesis of the teichoic acid wall of *S. aureus* [2,3]. Due to this action on the bacterial wall, clomiphene exhibits synergy with β-lactams in restoring methicillin-resistant *S. aureus* susceptibility [3]. In addition, tamoxifen was shown to be active against *E. faecium* and *S. aureus* in vitro and in *Galleria mellonella* and murine models of infections, respectively [2,4]. A previous study from our research group showed that tamoxifen played an essential role in regulating immune cell traffic after infection by Gram-negative bacilli (i.e., *Acinetobacter baumannii*, *Pseudomonas aeruginosa*, and *Escherichia coli*) in order to reduce the hyperinflammation caused by sepsis and the bacterial burdens in animal tissues and fluids [1].

As with other antimicrobial agents like colistimethate sodium [5], tamoxifen is a prodrug converted after liver passage to three major active metabolites: 4-hydroxytamoxifen (HTAM), endoxifen (ENDX), and N-desmethyltamoxifen (DTAM) [6]. However, their antibacterial activities against Gram-negative bacteria remain unknown.

In this study, we reported that tamoxifen decreased the development of infection in immunosuppressive mice for *A. baumannii* and *E. coli*, but not *P. aeruginosa*, lowering their concentrations in tissues and blood and increasing the mice survival. Although tamoxifen did not present bactericidal nor bacteriostatic effects against *A. baumannii*, *P. aeruginosa*, and *E. coli* in vitro, we show that tamoxifen metabolites exhibited antibacterial activity against *A. baumannii* and *E. coli*, suggesting that tamoxifen metabolism is actively involved in the therapeutic efficacy of tamoxifen in vivo.

## 2. Results

### 2.1. Tamoxifen Increases Mice Survival and Decreases the Bacterial Burden in Immunosuppressed Mice

Previous studies demonstrated that infection with *A. baumannii*, *P. aeruginosa*, and *E. coli* in immunosuppressed mice is lethal [7,8,9]. To determine whether tamoxifen treatment is therapeutically effective in immunosuppressed mice, we treated immunocompetent mice with cyclophosphamide to reduce circulating monocytes and neutrophils. After *A. baumannii* and *E. coli* infection in these immunosuppressed mice, tamoxifen treatment increased mouse survival in both groups to 66.67% (Table 1). However, with *P. aeruginosa*, survival was only 16.67% (Table 1). Bacterial loads of *A. baumannii* and *E. coli* in spleen, lung, and blood were reduced in both immunosuppressed and immunocompetent mice after treatment with tamoxifen. In contrast, bacterial loads of *P. aeruginosa* in tissues and blood were not reduced in immunosuppressed mice after treating with tamoxifen when compared with immunocompetent mice (Table 1). These findings suggest that tamoxifen helped clear *A. baumannii* and *E. coli* infections even though mice were immunosuppressed by an additional independent immune response mechanism.

### 2.2. Antibacterial Activity of Tamoxifen Metabolites

Despite the fact that tamoxifen has no bactericidal activity in vitro (MIC >256 mg/L) (Table 2), we reasoned that the in vivo antimicrobial activity of tamoxifen observed in immunosuppressed mice should result from tamoxifen metabolism in mice. Susceptibility assays showed that the tamoxifen metabolites mixture exhibited MIC values of 8 mg/L against the American Type Culture Collection (ATCC) reference strains of *A. baumannii* and *E. coli* (ATCC 17978 and ATCC 25922), and ≥64 mg/L against the reference strain of *P. aeruginosa* (PAO1). In the case of multidrug-resistant (MDR) strains of *A. baumannii* and *E. coli* (Ab186 and EcMCR+), the MIC of tamoxifen metabolites mixture was 4 and 32 mg/L, respectively (Table 2).

**Figure 1:**
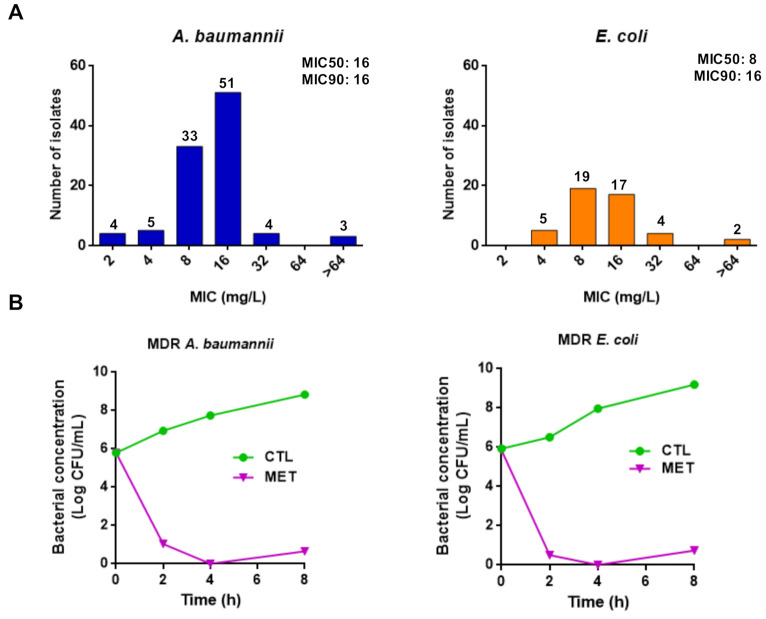
Tamoxifen metabolites present antibacterial activity against *A. baumannii* and *E. coli*. (**A**) Histogram distribution of MIC for the three tamoxifen metabolites mixture against a collection of *A. baumannii* and *E. coli*. (**B**) Time-kill curves of the multidrug-resistant (MDR) *A. baumannii* Ab186 and *E. coli* EcMCR+ strains alone and in the presence of metabolites mixture (4× MIC) for 8 h.

In addition, we extended the susceptibility assays against a clinical collection of *A. baumannii*, *P. aeruginosa*, and *E. coli*. We showed that the three tamoxifen metabolites mixture exhibited MIC_50_ values of 16 and 8 mg/L, and MIC_90_ values of 16 mg/L against 100 and 47 clinical strains of *A. baumannii* and *E. coli*, respectively (Figure 1A). In contrast, these metabolites mixture presented a MIC_50_ and MIC_90_ value of >64 mg/L against 24 clinical strains of *P. aeruginosa* (data not shown).

Using time-course assays, we examined the bactericidal activity of the three tamoxifen metabolites against MDR strains of *A. baumannii* and *E. coli* (Ab186 and EcMCR+). Figure 1B shows that 4× MIC of the three tamoxifen metabolites mixture demonstrated bactericidal effect during 8 h of growth, decreasing the bacterial count by >5 log_10_ colony forming unit (CFU)/mL compared with the initial bacterial inoculum. These data confirm the antibacterial activity of tamoxifen metabolites observed in microdilution assays.

### 2.3. Effect of Tamoxifen Metabolites on the Bacterial Cell Membrane

**Figure 2:**
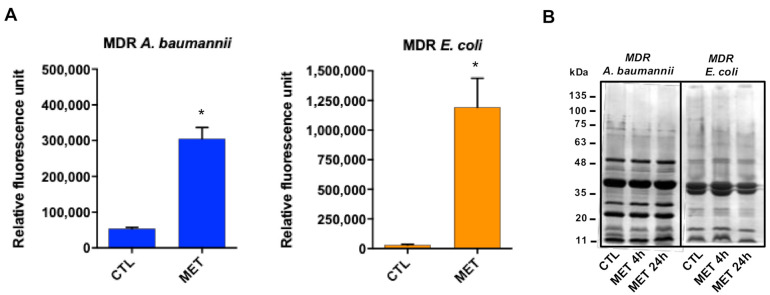
Tamoxifen metabolites present antibacterial activity targeting the bacterial membrane. (**A**) Tamoxifen metabolites effect on the bacterial permeability. The membrane permeabilization of MDR *A. baumannii* Ab186 and *E. coli* EcMCR+ strains in absence and presence of tamoxifen metabolites (2 and 16 mg/L, respectively) incubated for 24 h, was quantified by Typhon Scanner. (**B**) SDS–PAGE of the outer membrane proteins of MDR *A. baumannii* Ab186 and *E. coli* EcMCR+ strains with or without tamoxifen metabolites (2 and 16 mg/L, respectively). MET: the three tamoxifen metabolites together. CTL: control. * *p* < 0.05: CTL vs. MET.

In order to determine the mode of action of tamoxifen metabolites, we examined their effect on the membrane permeability. The three tamoxifen metabolites mixture strongly increased membrane permeability (Figure 2A) without affecting the outer membrane protein (OMP) profile (Figure 2B). This suggests that tamoxifen metabolites affect only the integrity of the bacterial cell wall without changing OMP expression.

## 3. Discussion

We herein provide the first evidence of tamoxifen antibacterial activity in vivo through the generation of active metabolites presenting bactericidal activity against *A. baumannii* and *E. coli*. Among the SERMs investigated, clomiphene and tamoxifen showed activity against *E. faecium* [2,3]. Previously, we reported that tamoxifen showed good activity against *A. baumannii*, *P. aeruginosa*, and *E. coli* infections through the regulation of immune cells traffic from bone marrow to blood after bacterial infection [1].

Tamoxifen therapeutic efficacy does not only regulate innate immune responses, but offers different responses depending on bacteria type. We demonstrated the excellent therapeutic efficacy of tamoxifen against *A. baumannii*, *P. aeruginosa*, and *E. coli*, even though this efficacy was slightly lower against *P. aeruginosa*. However, tamoxifen reduced the migration of immune cells from bone marrow to blood in mice infected by these three pathogens at similar levels [1]. A possible explanation could be the involvement of an additional independent immune response mechanism.

This hypothesis is in agreement with the results we obtained in this study in immunosuppressed mice, in which tamoxifen had therapeutic efficacy against *A. baumannii* and *E. coli*, but not *P. aeruginosa*. In addition, the three major active tamoxifen metabolites (i.e., DTAM, ENDX, and HTAM) present bactericidal activity in monotherapy against *A. baumannii* and *E. coli*, but not against *P. aeruginosa*, as a consequence of extensive metabolization via cytochrome P450 enzymes [6]. These results are consistent with a therapeutic efficacy of tamoxifen depending on antibacterial activity added to immune response mechanisms.

The defective activity of tamoxifen metabolites against *P. aeruginosa* could be due to the more hydrophobic outer membrane of *P. aeruginosa,* which might interfere with tamoxifen metabolites penetration. It is well-known that *P. aeruginosa* lack general porins and instead have a large number of substrate-specific channels for nutrient transport [10]. Due to the lack of porins, the outer membrane of *P. aeruginosa* is highly impermeable, making it resistant to many antibiotics [11].

It is important to mention that few studies have investigated the activity of tamoxifen metabolites against infectious agents, yet there has been no report on their activity against Gram-negative bacteria [12]. One of these metabolites, HTAM, presented activity when used in monotherapy against *Mycobacterium tuberculosis* (MIC_50_ 2.5–5 mg/L) and in combination with rifampin, isoniazid, and ethambutol buforin II, showing its highest activity at 10 and 20 mg/L [13]. In addition, HTAM was also reported to be active against *Plasmodium falciparum* and *Cryptococcus neoformans* var. grubii [14,15]. In turn, endoxifen presented activity against *C. neoformans* var. grubii with MIC of 4 mg/L [15].

In this study, we showed that the three tamoxifen metabolites together produced an increase in membrane permeability of MDR *A. baumannii* and *E. coli* without modifying OMP profiles. This indicates that increasing membrane permeability could not be related to the changes in OMP expression. It is well known that the mechanism of action of tamoxifen in fungi is related to calmodulin binding [16,17]. Additionally, Scott et al. showed that HTAM inhibited the phospholipase D in *P. aeruginosa* [18]. Future studies on the mechanism of action of tamoxifen metabolites against Gram-negative bacteria and on their therapeutic efficacy in animal experimental models of infection would be of interest.

## 4. Materials and Methods

### 4.1. Reagents

Tamoxifen, DTAM, ENDX, and HTAM, and porcine mucin were obtained from Sigma, Spain. Cyclophosphamide was obtained from Baxter, Spain.

### 4.2. Bacterial Strains

Reference strains of *A. baumannii* (ATCC 17978), *P. aeruginosa* (PAO1), and *E. coli* (ATCC 25922) were used [19,20,21]. We also used the clinical MDR strain of *A. baumannii* (Ab186) [22], 100 clinical strains of *A. baumannii* from the REIPI–GEIH 2010 collection [23], the clinical MDR strain of *E. coli* (EcMCR+) carrying *mcr-1* gene [24], and 47 clinical strains of *E. coli* from the Bact-OmpA collection [25].

### 4.3. Animals

Immunocompetent C57BL/6 female mice (16–18 g and 8 weeks of age) were obtained from the University of Seville. All mice had murine-pathogen-free sanitary status and were assessed for genetic authenticity and housed in regulation cages with food and water ad libitum. This study was carried out in strict accordance with the protocol approved by the Committee on the Ethics of Animal Experiments of the University Hospital of Virgen del Rocío, Seville (0704-N-18). All surgery was performed under sodium thiopental anesthesia and all efforts were made to minimize suffering.

### 4.4. Immunosuppressed Mice

Blood frequencies of monocytes and neutrophils were reduced with cyclophosphamide treatment following the protocol described by Zuluaga et al. [9]. Immunocompetent C57BL/6 female mice were treated with cyclophosphamide at 100 and 150 mg/kg at day 4 and 1, respectively, before bacterial infection.

### 4.5. Therapeutic Effect of Tamoxifen in Immunocompetent Murine Models of Peritoneal Sepsis

Immunocompetent murine peritoneal sepsis models of *A. baumannii* (ATCC 17978), *P. aeruginosa* (PAO1), and *E. coli* (ATCC 25922) strains were established via intraperitoneal inoculation of bacteria in immunocompetent mice [26]. Briefly, 6 animals of each group were infected intraperitoneally with 0.5 mL of the MLD100 of ATCC 17978 (3.2 log_10_ CFU/mL), PAO1 (4.9 log_10_ CFU/mL), and ATCC 25922 (4.7 log_10_ CFU/mL) mixed 1:1 with 10% porcine mucin. Tamoxifen therapy was administered for 3 days at one safe dose of 80 mg/kg/d using corn oil as a vehicle before bacterial inoculation [4,27]. Mice were randomly ascribed to the following groups: 1) controls (without treatment), and 2) tamoxifen administered at 80 mg/kg/d intraperitoneally (i.p.) for three days before the bacterial inoculation of each strain. Mortality was recorded over three days. After the death or sacrifice of mice, aseptic thoracotomies were performed, and blood samples were obtained by cardiac puncture. The spleen and lungs were aseptically removed and homogenized (Stomacher 80; Tekmar Co., Vernon, BC, Canada) in 2 mL of sterile NaCl 0.9% solution. Next, 10-fold dilutions of the homogenized spleen, lungs, and blood were plated onto sheep blood agar (Becton Dickinson Microbiology Systems, Franklin Lakes, NJ, USA) for quantitative cultures. Plates were incubated for 24 h at 37 °C, and after colony counts, the log_10_ value of viable cells (CFU/g or mL) was determined. If no growth was observed after plating the whole residue of the homogenized tissue and blood, a logarithm value corresponding to the limit of detection of the method (1 CFU) was assigned.

### 4.6. Therapeutic Effect of Tamoxifen in Immunosuppressed Murine Models of Peritoneal Sepsis

The immunosuppressed murine peritoneal sepsis models of *A. baumannii* (ATCC 17978), *P. aeruginosa* (PAO1), and *E. coli* (ATCC 25922) strains were established via intraperitoneal inoculation of bacteria in immunosuppressed mice. Briefly, animals (6 mice for each group) were infected intraperitoneally with 0.5 mL of the MLD100 of each strain mixed 1:1 with 10% porcine mucin. Tamoxifen therapy, mortality, and bacterial loads in tissues and blood were determined, as described in a previous section.

### 4.7. In Vitro Susceptibility Testing and Time-Kill Experiments

The MICs of tamoxifen, DTAM, ENDX, HTAM, and the mixture of three tamoxifen metabolites in equal concentrations against *A. baumannii*, *P. aeruginosa*, and *E. coli* clinical strains were determined via a microdilution assay in two independent experiments, in accordance with CLSI guidelines [28].

Time-kill kinetic assays of the MDR *A. baumannii* Ab186 and MDR *E. coli* EcMCR+ strains were conducted in Mueller–Hinton broth in the presence of the mixture of the three tamoxifen metabolites at 4× MIC and were performed in duplicate as previously described [28]. Drug-free broth was evaluated in parallel as a control and cultures were incubated at 37 °C. Viable counts were determined by serial dilution at 0, 2, 4, and 8  h after adding the three tamoxifen metabolites, and plating 100  μL of control, test cultures, or dilutions at the indicated times onto sheep blood agar plates. Plates were incubated for 24 h and, after colony counts, the log_10_ value of viable cells (CFU/mL) was determined.

### 4.8. Analysis of Outer Membrane Proteins (OMPs) by SDS–PAGE

Bacterial cells of MDR strain of *A. baumannii* Ab186 and MDR strain of *E. coli* EcMCR+ were grown in Luria-Bertani (LB) broth in the logarithmic phase, incubated with 2 and 16 mg/L of tamoxifen metabolites mixture, respectively, for 4 or 24 h, and lysed by sonication. OMPs were extracted with sodium lauroyl sarcosinate (Sigma, Spain) and recovered by ultracentrifugation, as described previously [29]. OMP profiles were determined via sodium dodecyl sulfate polyacrylamide gel electrophoresis (SDS–PAGE) using 10% SDS gels and 6 μg protein of OMPs, followed by SimplyBlue SafeStain gel (Invitrogen, Spain).

### 4.9. Membrane Permeability Assays

Bacterial suspensions (adjusted to optical density at 600 nm = 0.2) of MDR *A. baumannii* Ab186 and MDR *E. coli* EcMCR+ strains were placed on a 96-well plate, incubated with 2 and 16 mg/L of tamoxifen metabolites mixture, respectively, and mixed in a solution of phosphate buffered saline containing Ethidium Homodimer-1 (EthD-1) (1:500) (Invitrogen, Carlsbad, CA, USA). After 10 min of incubation, fluorescence was monitored during 160 min using a Typhoon FLA 9000 laser scanner (GE Healthcare Life Sciences, Marlborough, MA, USA) and quantified with ImageQuant TL software (GE Healthcare Life Sciences, USA). Bacterial counts were obtained at the beginning and end of the experiment to ensure that the metabolite mixture did not present bactericidal activity against *A. baumannii* and *E. coli* strains.

### 4.10. Statistical Analysis

Group data were presented as means  ±  standard errors of means (SEM). Difference in membrane permeability were assessed by Student *t*-test. Differences in bacterial spleen, lung, and blood concentrations (mean ± SEM log_10_ CFU per g or mL) were assessed by analysis of variance (ANOVA) and post-hoc Dunnett and Tukey tests. Differences in mortality (%) between groups were compared using the χ2 test. *p* values of <0.05 were considered significant. The SPSS (version 21.0; SPSS Inc., Armonk, NY, USA) statistical package was used.

## 5. Conclusions

The results of this study indicated that tamoxifen metabolites were active against MDR *A. baumannii* and *E. coli* and might be potential antimicrobial agents to treat infections by these pathogens.

## Figures and Tables

**Table 1:** Tamoxifen shows therapeutic efficacy in immunocompetent and immunosuppressed murine models of peritoneal sepsis by Gram-negative bacilli.

Strain	Treatment	*n*	Bacterial Load (log CFU/g or mL ± SEM)			Survival (%)
			Spleen	Lung	Blood	
*A. baumannii* ATCC 17978	CTL	6	9.51 ± 0.17	9.77 ± 0.17	6.14 ± 0.94	0
	CPM	6	10.06 ± 0.24	9.91 ± 0.25	8.32 ± 0.25 ^a^	0
	TAM	6	2.87 ± 1.21	2.61 ± 1.07	0.61 ± 0.61	100
	CPM + TAM	6	3.14 ± 1.25^c^	3.46 ± 1.31^c^	2.33 ± 1.00 ^c^	66.7
*P. aeruginosa* PAO1	CTL	5	8.91 ± 0.15	9.24 ± 0.17	6.71 ± 0.27	0
	CPM	6	9.94 ± 0.06 ^a^	9.36 ± 0.13	7.09 ± 0.28	0
	TAM	6	5.33 ± 1.08	4.14 ± 1.50	1.26 ± 1.26	66.7
	CPM + TAM	6	8.71 ± 0.94 ^b^	8.77 ± 0.83 ^b^	4.04 ± 0.82 ^b,c^	16.7
*E. coli* ATCC 25922	CTL	6	8.71 ± 0.05	8.88 ± 0.16	8.18 ± 0.37	0
	CPM	6	10.55 ± 0.13 ^a^	9.92 ± 0.19 ^a^	6.92 ± 0.38^a^	0
	TAM	6	5.01 ± 1.20	4.72 ± 1.08	3.87 ± 0.99	83.3
	CPM + TAM	6	5.93 ± 1.22 ^c^	5.92 ± 1.00 ^c^	3.95 ± 1.22 ^c^	66.7

^a^*p* < 0.05: bacteria vs. bacteria + CPM, ^b^ *p* < 0.05: bacteria + TAM vs. bacteria + CPM + TAM, ^c^ *p* < 0.05: bacteria + CPM vs. bacteria + CPM + TAM. ATCC: American Type Culture Collection, CPM: cyclophosphamide, TAM: tamoxifen, CFU: colony forming unit, CTL: control, SEM: standard error median.

**Table 2:** Minimal inhibitory concentrations of tamoxifen and its metabolites for *A. baumannii* ATCC 17978, *P. aeruginosa* PAO1, and *E. coli* ATCC 25922 strains.

Strain	TAM (mg/L)	DTAM (mg/L)	ENDX (mg/L)	HTAM (mg/L)	MET (mg/L)
*A. baumannii* ATCC 17978	>256	8	16	16	8
*A. baumannii* Ab186	>256	16	16	32	4
*P. aeruginosa* PAO1	>256	≥64	≥64	≥64	≥64
*E. coli* ATCC 25922	>256	8	16	32	8
*E. coli* EcMCR^+^	>256	≥64	64	≥64	32

ATCC: American Type Culture Collection, TAM: tamoxifen, HTAM: 4-hydroxytamoxifen, DTAM: N-desmethyltamoxifen, ENDX: endoxifen, TAM: 4-hydroxytamoxifen, MET: mixture of the three tamoxifen metabolites.

## Data Availability

Not applicable.
